# Estimating the cost-effectiveness profile of a universal vaccination programme with a nine-valent HPV vaccine in Austria

**DOI:** 10.1186/s12879-016-1483-5

**Published:** 2016-04-16

**Authors:** L. Boiron, E. Joura, N. Largeron, B. Prager, M. Uhart

**Affiliations:** Sanofi Pasteur MSD, 162 avenue Jean Jaurès CS 50712 69367, Lyon, Cedex 07 France; Department of Gynecology and Obstetrics, Medical University of Vienna, Comprehensive Cancer Center, Vienna, Austria; Sanofi Pasteur MSD, Campus 21, Europarin F11/402, Brunn am Gebirge, A-2345 Austria

**Keywords:** Cost-effectiveness, Austria, HPV, Cervical cancer, Vaccination

## Abstract

**Background:**

HPV is a major cancer-causing factor in both sexes in the cervix, vulva, vagina, anus, penis, oropharynx as well as the causal factor in other diseases such as genital warts and recurrent respiratory papillomatis. In the context of the arrival of a nonavalent HPV vaccine (6/11/16/18/31/33/45/52/58), this analysis aims to estimate the public health impact and the incremental cost-effectiveness of a universal (girls and boys) vaccination program with a nonavalent HPV vaccine as compared to the current universal vaccination program with a quadrivalent HPV vaccine (6/11/16/18), in Austria.

**Method:**

A dynamic transmission model including a wide range of health and cost outcomes related to cervical, anal, vulvar, vaginal diseases and genital warts was calibrated to Austrian epidemiological data. The clinical impact due to the 5 new types was included for cervical and anal diseases outcomes only. In the base case, a two-dose schedule, lifelong vaccine type-specific protection and a vaccination coverage rate of 60 % and 40 % for girls and boys respectively for the 9-year old cohorts were assumed. A cost-effectiveness threshold of €30,000/QALY-gained was considered.

**Results:**

Universal vaccination with the nonavalent vaccine was shown to reduce the incidence of HPV16/18/31/33/45/52/58 -related cervical cancer by 92 %, the related CIN2/3 cases by 96 % and anal cancer by 83 % and 76 % respectively in females and males after 100 years, relative to 75 %, 76 %, 80 % and 74 % with the quadrivalent vaccine, respectively. Furthermore, the nonavalent vaccine was projected to prevent an additional 14,893 cases of CIN2/3 and 2544 cases of cervical cancer, over 100 years. Depending on the vaccine price, the strategy was shown to be from cost-saving to cost-effective.

**Conclusion:**

The present evaluation showed that vaccinating 60 % of girls and 40 % of boys aged 9 in Austria with a 9-valent vaccine will substantially reduce the incidence of cervical cancer, CIN and anal cancer compared to the existing strategy. The vaccination strategies performed with the 9-valent vaccine in the current study were all found to be cost-effective compared to the current quadrivalent vaccination strategy by considering a cost-effectiveness threshold of 30,000€/QALY gained.

## Background

The Human Papillomavirus (HPV) is a virus that infects squamous epithelia [1]. HPV is the most common sexually transmitted infection and can be passed on through genital contact or by skin-to-skin contact [2, 3]. HPV infections are most of the time transient and cleared up within a few months after acquisition. However, in some cases, HPV infection can persist and progress to non-cancerous or cancerous lesions [4]. HPV is recognized as a major cancer causing factor in both sexes: in the cervix, vulva, vagina for females, in the penis for males and in the anus and oropharynx in both sexes, as well as the causal factor of other diseases such as genital warts and recurrent respiratory papillomatis (mainly due to HPV 6 and 11). The HPV-related burden is substantial for individuals, healthcare systems and society as a whole. Cervical cancer is the second most common female cancer in women aged 15 to 44 in the European Union, and nearly all cases can be attributed to HPV infection [5, 6]. In Europe, it is estimated that about 34 thousands new cervical cancer cases are diagnosed and 13 thousands new cervical cancer deaths occurred in 2012 [4]. Two preventative strategies are used in combination to avert cervical cancer: the screening (secondary prevention) and the HPV vaccination (primary prevention). The screening allows to detect – and then treat - precancerous lesions before they evolve into cancer. The screening is very effective but is only implemented for cervical cancer prevention. No screening exists for other HPV cancers. In Austria, the cytology screening is opportunistic: Participation depends on the individual decision.

There are currently two commercially available vaccines in Europe: the quadrivalent (HPV 6, 11, 16, 18) vaccine Gardasil® and the bivalent (HPV 16, 18) vaccine Cervarix®. A new generation of vaccine, the nona-valent vaccine, has been recently approved by the EMA [7, 8]. This new vaccine will expand coverage against 5 more oncogenic types (HPV 31, 33, 45, 52, 58) in addition to the 4 original types included in Gardasil®. Indeed, the nonavalent vaccine has the potential to prevent approximately 90 % of cervical, vulvar, vaginal and anal cancers and 80 % of precancerous lesions [9, 10].

Since 2008, HPV vaccine programmes have been implemented in most EU countries in girls. Austria is the first country in Europe having implemented a HPV vaccination programme for girls and boys in 2014. Outside Europe, a universal vaccination programme is implemented in the US, some Canadian provinces, in Australia and in Israel. Economic and social considerations associated with male vaccination have been widely discussed [11–15]. Overall, extension of the vaccination program to males is mainly justified by (i) epidemiological reasons: the burden of HPV related diseases in boys and men is substantial, (ii) equity reasons: men who have sex with men do not benefit from the herd immunity conferred by girls’ vaccination, and (iii) efficiency reasons: herd protection can only be achieved if the vaccination program has a sufficiently coverage rate. Vaccinating boys and girls is a way to improve the coverage and to stop the spread of the associated diseases [16, 17].

In the context of the recent availability of the nonavalent HPV vaccine, and considering the heavy HPV-related economic burden imposed on healthcare systems and society, it is important to inform policy and decision makers on the expected public health impact and cost-effectiveness of the new vaccine compared to existing preventive strategies.

In Austria as well as other countries in Europe, numerous are the published studies assessing the cost-effectiveness of HPV vaccines [18–21]. So far, however, there has not been any analysis assessing the cost-effectiveness of the nonavalent vaccine in Europe. On the contrary, the cost-effectiveness of nonavalent HPV vaccination was assessed in the United States, through 3 models which were presented during the Advisory Committee on Immunization Practices (ACIP) meeting in February 2015 [22–25]. Results were consistent across the three different models: universal vaccination with the nonavalent vaccine priced moderately was estimated to be cost-saving compared to universal vaccination with Gardasil®, or at least very cost-effective at higher prices.

The present analysis aimed to assess the incremental public health impact and to provide realistic cost-effectiveness estimates of a girls and boys (universal) vaccination program with the nonavalent HPV vaccine compared to the current universal quadrivalent HPV vaccine, in Austria, both performed in conjunction with the current screening strategy.

## Methods

### Mathematical model

A previously published US model, simulating the natural history of genotypes 6, 11, 16, 18 HPV-infections and estimating the cost associated with HPV-related diseases, has been extended to account for infections and diseases attributable to HPV genotypes 31, 33, 45, 52, 58 and adapted to Austria in order to estimate the cost-effectiveness of a 9-valent vaccine against Human Papillomavirus [26–29].

The model consists of three connected modules: (1) a demographic model that defines the demographic characteristics of the population and describes how persons enter, age within, and exit the model; (2) an epidemiologic module that simulates HPV transmission and the occurrence of HPV-related diseases; (3) an economic model that estimates costs and quality of life associated with the screening, vaccination and management of the disease for each prevention strategy. A detailed description of the model was presented in Elbasha et al. [29].

The epidemiologic module is constituted of 16 separate and independent models to take into account the many HPV-types and many diseases related to. Whereas HPV 6, 11, 16 and 18 are modelled separately, the 5 additional types are combined into a single set of compartments. Indeed, the epidemiologic module includes one HPV6-specific model (RRP, genital warts and CIN1) one HPV11-specific model (RPP and genital warts), one separate model for each disease related to HPV 16 or HPV18 (cervical precancerous lesion and cancer, vulvar precancerous lesion and cancer, vagina precancerous lesions and cancer, anal precancerous lesion and cancer, penile precancerous lesions and cancer, and head and neck cancer). Last, the 5 additional types (HPV31, 33, 45, 52 and 58) have been merged as one “super-type” for which one separate model has been created for cervical diseases and another one for anal diseases. All together, the model accounts for the transmission dynamics of nine HPV types: 16, 18, 6, 11, 31, 33, 45, 52, and 58, and simulate the occurrence of genital warts; RRP; pre-cancers such as cervical intraepithelial neoplasia (CIN); cervical, vulvar, vaginal, penile, anal, and head/neck cancers related to these HPV types. The current analysis assumes only cervical cancers and pre-cancers and anal cancers have significant contributions from the 5 additional types. The contribution of the types 33/33/45/52/58 to the burden of other diseases (vaginal, vulvar, penile, head and neck cancers, genital warts, and RRP) was not modeled.

Different strategies were analysed and compared:

- 9-valent vaccine for girls and boys associated with current cervical cancer screening;
- 4-valent vaccine for girls and boys associated with current cervical cancer screening.

### Input parameters

#### Demographics & sexual behavior

Population data was retrieved from Statistics Austria. The total population in Austria at the beginning of the year 2014 was estimated to be 8,507,786 people [30]. A constant population size was assumed. Data on sexual behaviour in Austria were scarce so the results from the UK NATSAL-3 study were used as they were deemed to be applicable to the Austrian setting according to expert opinion [31].

#### Natural history of disease and treatment patterns

The progression from infection to disease follows a similar natural history structure as the initial US model, previously described and reported [29]. As transmission rates are not directly observable, calibration techniques were used to obtain the best set of parameters.

The female population receiving hysterectomy over the course of 1 year by age group was first estimated from the incidence rates of hysterectomy by age reported by the German Statistical Office [32]. Then, rates have been adjusted to the number of hysterectomy cases in Austria reported by Statistics Austria (2013) [33].

#### Screening

The annual cervical cancer screening rates were extracted from the report by Zechmeister [19]. The percent of females receiving gynecological cancer screening tests at least once every 3 years was set to 47 % [34] and the percentage of women receiving a follow-up screening test after abnormal Papanicolaou test (Pap test or Pap smear) was estimated at 90 % according to expert opinion. Since no screening program for vulvar and vaginal cancer screening practice exists, the percentage of females receiving regular vaginal cancer screening was set to 0 %.

#### Vaccination strategy

The current vaccination program in Austria is for girls and boys to get vaccinated at their 9^th^ year of age, with vaccination coverage rates assumed of 60 % for girls and 40 % for boys [35]. The vaccination consists of a two-dose schedule assuming an adherence rate (proportion receiving the 2^nd^ dose) of 100 %. It was deemed that universal vaccination with the nonavalent vaccine would have the same characteristics (coverage and adherence) as the current vaccination for girls and boys and that performance does not differ between the two vaccines.

#### Vaccine properties

The prophylactic efficacy of the vaccine or vaccine-conferred degree of protection was based on clinical trial data (Table 1) [36–41]. The duration of protection against HPV genotypes 6/11/16/18/31/33/45/52/58 was assumed to be lifelong. This assumption relies on the immunogenicity and effectiveness data of Gardasil® [42–44] that have demonstrated an efficacy up to ten years and the mathematical modelling of antibody decay following vaccination. Indeed, the long term duration of protection afforded by HPV vaccination was modelled on data obtained from phase II study involving a monovalent HPV 16 vaccine. The predicted persistence of anti-HPV levels over time was estimated using two mixed effects models. The first was a conventional model of antibody decay and the second was a modified model that accounts for long-lived immune memory. Using the antibody decay model, it was estimated that following administration of a three-dose regimen of HPV-16 vaccine [45] in women aged 16–23 years, anti-HPV-16 levels will remain above those induced naturally by HPV-16 infection for 12 years, and above detectable levels for 32 years in 50 % of vaccinees. With the modified model, which fitted the data better (*p* < 0.001), it was estimated that near life-long persistence of anti-HPV-16 following vaccination is expected in 99 % of subjects. Given that each of the nine VLPs contained in Gardasil 9 are the relevant L1 protein and all are therefore expected to elicit an immune response by the same mechanism, there is currently no evidence to suggest that similar long term duration of protection would not apply to HPV types 6, 11, 18, 31, 33, 45, 52 and 58. Duration of protection was also tested in sensitivity analyses with lower duration of 20 years.

**Table 1:** Summary table on vaccine efficacy

Vaccine assumptions	HPV 16	HPV 18	HPV 31, 33, 45, 52 and 58
Cervical cancer			
Vaccine efficacy for preventing cervical HPV16/18/31/33/45/52/58 infections:			
- Male^a^	0.411	0.621	0.411
- Female^b^	0.76	0.963	0.76
Degree of protection of the vaccine against cervical HPV16/18 infections becoming persistent	0.988	0.984	0.988
Degree of protection of the vaccine against HPV16/18 -related CIN	0.979	1	0.979
Vaginal and vulvar cancers			
Vaccine efficacy for preventing vaginal/vulvar HPV16/18 infections:			
- Male^a^	0.411	0.621	
- Female^b^	0.76	0.963	
Degree of protection of the vaccine against vaginal/vulvar HPV16/18 infections becoming persistent	0.988	0.984	
Degree of protection of the vaccine against HPV16/18-related /VaIN/VIN	1	1	
Anal cancers			
Vaccine efficacy for preventing anal infections			
- Male^a^	0.411	0.621	0.621
- Female^b^	0.76	0.963	0.963
Degree of protection of the vaccine against anal infections becoming persistent			
- Male^a^	0.787	0.96	0.96
- Female^b^	0.988	0.984	0.984
Degree of protection of the vaccine against HPV16/18 -related AIN neoplasia	0	0	0
Penile and H&N cancers			
Vaccine efficacy for preventing penile and H&N infections			
- Male^a^	0.411	0.621	
- Female^b^	0.76	0.963	
Degree of protection of the vaccine against penile and H&N infections becoming persistent			
- Male^a^	0.787	0.96	
- Female^b^	0.988	0.984	
Degree of protection of the vaccine against HPV16/18 -related PIN and H&N neoplasia	0	0	

^a^Preventing male genital infections through male vaccination is assumed to prevent transmission of genital infections to females

^b^Preventing female genital infections through vaccination is assumed to prevent transmission of genital infections to males

Source: Giuliano et al. (2011) [38] and Elbasha and Dasbach (2010) [28]

As the model includes degree of protection against infection and degree of protection against disease arising from a breakthrough infection, it considers different efficacy values against infection and against disease. In the model, it is further assumed that these “breakthrough” infections are transmissible. The efficacy against anal, head and neck, penile and RRP diseases was assumed to be conferred through protection against infection only.

Values on vaccine efficacy were not available specifically for the Austrian population. The US model has been already shown to be transferable to other countries [46]. In line with the current Austrian recommendation, a two-dose regimen was considered in the model for quadrivalent vaccine and also for 9-valent vaccine.

As the duration and strength of effectiveness of cross protection is uncertain [47–51] and has still to be demonstrated as highlighted in the recent WHO guidance on cervical cancer, no cross-protection was assumed in the base case [52].

Vaccine efficacy parameters considered in the model are presented in the Table 1.

#### Perspective

In Austria, HPV vaccination is delivered through a national public programme and vaccine purchase is realized with public tenders. All the costs are from the perspective of the payer.

#### Cost of vaccination

In Austria, the manufacturer’s price of the quadrivalent vaccine is 110 € [21]. The price of the nonavalent vaccine was not available in Austria since the product is not yet marketed. A broad range of prices for the nonavalent vaccine was assessed, from 110 € up to the maximum cost-effective price. A theoretical price of 135 € for the nonavalent vaccine was assumed in the base case and for the sensitivity analysis. It corresponds to the average of the price in the private sector (147.91 €^*^^1^) and the CDC price (121.76 €^*1^) of the nonavalent vaccine currently observed in the US [53]. The administration cost per dose was set at 12 € [18].

#### Cost per episode of care

The costs per episode of care of each HPV-related disease, defined as the cost of management from diagnosis to resolution of the case are reported in the Table 2. Costs were retrieved from Hillemanns et al. [54], Hampl et al. [55], Zechmeister et al. [19], Brisson et al. (2013) [22], and Jit et al. [56]. The productivity losses as a result of HPV disease were not included in the model.

**Table 2:** Summary table on costs and utilities for HPV-related disease

Parameter	Gender	Inflated values (€2014)	Utility [27, 58, 59]
- CIN 1	Female	377 € [54]	0.91
- CIN 2	Female	377 € [54]	0.87
- CIN 3 and CIS	Female	1681 € [54]	0.87
- Cervical cancer, local disease^a^	Female	19,151 € [19]	0.76
- Cervical cancer, regional disease^a^	Female	31,978 € [19]	0.67
- Cervical cancer, distant disease^a^	Female	32,651 € [19]	0.48
- VaIN 2	Female	936 € [55]	0.87
- VaIN 3, CIS	Female	2766 € [55]	0.87
- Vaginal cancer, local disease^a^	Female	16,661 € [19]	0.76
- Vaginal cancer, regional disease^a^	Female	27,820 € [19]	0.67
- Vaginal cancer, distant disease^a^	Female	28,406 € [19]	0.48
- Vulvar cancer, local disease^a^	Female	16,661 € [19]	0.76
- Vulvar cancer, regional disease^a^	Female	27,820 € [19]	0.67
- Vulvar cancer, distant disease^a^	Female	28,406 € [19]	0.48
- Penile cancer, local disease^a^	Male	13,597 € [19]	0.76
- Penile cancer, regional disease^a^	Male	22,703 € [19]	0.67
- Penile, distant disease^a^	Male	23,182 € [19]	0.48
- Anal cancer, local disease^a^	Male; Female	19,534 € [19]	0.76
- Anal cancer, regional disease^a^	Male; Female	32,617 € [19]	0.67
- anal cancer, distant disease^a^	Male; Female	33,304 € [19]	0.48
- Head & Neck cancer, local disease^a^	Male; Female	22,981 € [19]	0.76
- Head & Neck cancer, regional disease^a^	Male; Female	38,373 € [19]	0.67
- Head & Neck cancer, distant disease^a^	Male; Female	39,181 € [19]	0.48
- Genital warts	Male; Female	661 € [54]	0.91
- Recurrent respiratory papillomatosis	Male; Female	26,812 € [56]	0.81

^a^Disease stages can be related to the traditional Tumour-Node-Metastasis (TNM) classification system as followed: − “Local disease” corresponds to stages I and II TNM classification, i.e., localized primary tumour; “Regional disease” corresponds to stage III TNM classification system, i.e., metastasis to regional lymph nodes; “Distant disease” corresponds to stage IV TNM classification system, i.e., distant metastatic disease

#### Cost of screening and diagnostic tests

A previous Austrian cost-effectiveness analysis of Zechmeister et al. (2007) was used to extract the costs of PAP test, colposcopy and biopsy [20]. Screening by PAP smear was set at 23 €, colposcopy cost at 9 € and Biopsy cost at 28 €.

All model costs were updated to 2014 Euros using the Consumer price indices in Austria, which was not specific for medical care [57].

#### Health-related quality of life

In the absence of Austrian-specific stage-stratified utility data in the population with HPV-related diseases, US data were used. The same utilities were used in the previous evaluations of the quadrivalent vaccine including the one conducted in Austria.

Health utility values for localized and regional cancer were estimated by Myers et al. [58] whereas Gold et al. [59] derived theses values for distant cancer. The other values were assumed by Elbasha et al. [27]. Utility values considered in the model are reported in the Table 2.

#### Discounting

In the absence of official health economic recommendation in Austria, discount rates of 3 % for both costs and benefits as reported in the latest economic evaluation performed in the country were used [18]. Alternative discount rates of 1 % and 4 % were tested in the sensitivity analysis.

#### Time horizon

An analytic horizon of 100 years was chosen because this was consistent with the time frame from which the system approached a steady state and the majority of the benefits and costs of vaccination could be realized as recently recommended by the European Vaccine Economics Community [60]. This time horizon is in accordance with the ones considered in the other evaluations of the nonavalent vaccine (100 years in Chesson et al. [23]; 70 years in Brisson et al. [22]).

### Model calibration and validation

The model was calibrated on incidence and mortality rates of HPV-related diseases observed in Austria.

The calibration process involved many rounds of iterations to move model outcomes closer to the targets. The following model outcomes were compared against the calibration target in each iteration: cervical cancer incidence, genital warts incidence, vaginal/vulvar/penile/anal/ head and neck cancer incidence, and mortality rates of cervical/vaginal/vulvar/ head and neck cancer.

The variables that affect all or most of the outputs are referred as global variables. These include behavioural parameters, natural history of disease, transmission rates, all-cause mortality and were first adjusted by changing transmission rates. The variables that affect only specific outputs are referred to as specific variables. These include probability of death and rate of seeking treatment for most cancers and were used to fine-tune each disease area.

#### Epidemiological targets

Regarding epidemiological data, incidental cases of cancers, Austrian-specific data were retrieved [61]. No Austrian data was available for cervical precancerous lesion incidences. Norwegian incidence data was used, as it was the lowest incidence among 5 different countries in Europe [4]. The incidence and mortality rates for the different cancers, and the incidence rate for genital warts, were adjusted by the proportion of diseases attributable to HPV infection and HPV genotype (Table 3) [4].

**Table 3:** Proportions of cancers and genital warts attributable to HPV infection

	Female		Male	
	4-valent vaccination	9-valent vaccination	4-valent vaccination	9-valent vaccination
Cervical cancer	72.8 %	89.0 %	–	–
Vaginal	50.7 %	60.6 %	–	–
Vulvar	14.2 %	16.2 %	–	–
Anal	76.3 %	78.7 %	76.3 %	78.7 %
Penile	–	–	34.4 %	34.4 %
Head and Neck	13.6 %	13.6 %	16.5 %	16.5 %
Genital warts	90 %	90 %	90 %	90 %

Source: Hartwig (2015) [4]

The mortality associated with HPV-related cancers was estimated from EUROCARE-5 survival data [62]. EUROCARE (European Cancer Registry) is a collaborative research project on cancer survival in Europe provided by 116 Cancer Registries in 30 European countries over the period 1999–2007. Since data from EUROCARE-5 was available from 15 years old, cancer-associated mortality rates were assumed to be 0 for the population below 15. As no relative survival by stage was found for Austria, a relative risk for local, regional and distant cancer was calculated according to a Cancer research in the UK [63]. Final mortality rates by stage and by age were obtained by multiplying each mortality rate by the corresponding relative risk.

Overall incidence and mortality by disease are reported in the Table 4.

**Table 4:** Overall incidence of cancers, mortality and genital warts

	Overall incidence (per 100.000)		Overall mortality (per 100.000)	
	Female	Male	Female	Male
Cervical	8.4	–	4.1	–
CIN 1	303.0	–	–	–
CIN 2+	138.8	–	–	–
Vaginal	1.1	–	0.2	–
Vulvar	3.1	–	0.9	–
Anal	1.8	1.0	0.3	0.3
Oral cavity	3.9	7.7	0.8	2.5
Larynx	0.9	9.1	0.3	1.6
Head & Neck	7.1	25.6	1.9	7.5
Genital warts	141.2	146.5	–	–
Penile	–	1.2	–	0.3

Source: Hartwig (2015) [4], ICO – Austria (2015) [61], Cancer research UK [63], Robert Koch Institute (2014) [74] and internal data for H&N and genital warts

### Model analyses

The model was used to estimate the total number of disease events related to the HPV type 6/11/16/18/31/33/45/52/58-related; the incidence and mortality of cervical cancer, anal cancer and the incidence of CIN and genital warts; the costs of vaccination, screening, diagnosis and management of the disease; the quality-adjusted life years (QALYs) of the model population. Results were reported over 100 years for the different strategies tested. Incremental cost-effectiveness ratios (ICERs) were then calculated by dividing the difference in accumulated costs by the QALY gained.

The interpretation of the incremental cost-effectiveness ratio of nonavalent vaccination in Austria is difficult in the absence of a formal cost-effectiveness threshold. However, international cost-effectiveness thresholds can help to estimate a realistic threshold for Austria:

- in the UK, the JCVI defined the cost-effectiveness threshold for vaccines is between 20,000 and 30,000 £/QALY (equivalent to 30,000 to 40,000 €/QALY in Austria).
- the WHO considers an intervention as very cost-effective if the ICER is below 1 GDP per capita (40,000 € for Austria) and cost-effective from 1 to 3 GDP per capita.

Thus it will be assumed that the cost effectiveness threshold in Austria is in the range from 30,000 € to 40,000 € per QALY gained.

Sensitivity analyses were performed deterministically, modifying the value of one base case parameter at a time. The following key parameters were tested: duration of protection (20 years), utilities (from Sullivan et al.), discount rates (1 % or 4 % for both health and outcomes), cost of diseases (+/− 50 %), variation in the VCR of boys and girls (+/− 20 %), increased vaccination coverage rate in boys (+10 %) and decreased compliance rate (90 % - assuming no efficacy if only one dose is administered). No probabilistic sensitivity analysis has been performed.

## Results

### Calibration

The calibration on overall incidence and mortality was good on a majority of the calibrated diseases (cervical, anal, penile, head and neck cancers and genital warts) as reported in Table 5.

**Table 5:** Comparison of overall incidence (/100,000) between target and calibration

	4-valent vaccination		9-valent vaccination	
	Target	Calibration results	Target	Calibration results
Female				
*Incidence*				
Cervical cancer	6.12	6.12	7.48	7.48
CIN 2+	63.15	22.29	114.23	27.62
Vaginal	0.56	0.05	0.67	0.05
Vulvar	0.44	0.08	0.50	0.08
Anal	1.37	1.37	1.42	1.42
Genital warts	127.08	126.93	127.08	126.93
Head and Neck	0.96	0.96	0.96	0.96
*Mortality*				
Cervical cancer	2.98	2.98	3.65	3.65
Vaginal	0.20	0.02	0.10	0.02
Vulvar	0.90	0.03	0.15	0.03
Anal	0.23	0.23	0.24	0.24
Male				
*Incidence*				
Penile cancer	0.41	0.41	0.41	0.41
Anal cancer	0.76	0.76	0.79	0.79
Genital warts	131.85	131.52	131.85	131.52
Head and Neck	4.23	4.25	4.23	4.25
*Mortality*				
Penile cancer	0.10	0.10	0.10	0.10
Anal cancer	0.23	0.22	0.24	0.23

However, a calibration on cervical precancerous was achieved but calibration results could not fit with the targets, producing really low values. For the diseases non-calibrated (vaginal and vulvar cancer), the model also reports lower values than expected. As a consequence, the model underestimates the benefits of the HPV vaccines on the cervical precancerous lesions and vaginal and vulvar cancers.

**Fig. 1:**
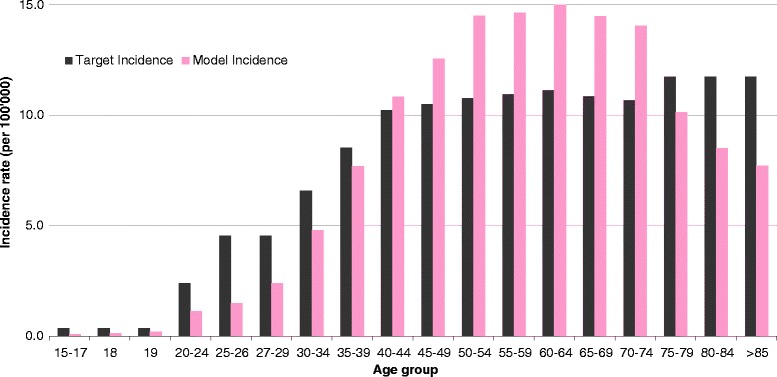
Calibration of cervical cancer HPV incidence related to types 16, 18, 6, 11, 31, 33, 45, 52, and 58

As the non-age specific calibration, the age-specific calibration shows good fit to the epidemiological data for the calibrated diseases (anal cancer, cervical cancer, genital warts) and head & neck cancers but underestimate the incidence of CINs, and vulvar and vaginal cancers (Fig. 1).

### Health outcomes

**Fig. 2:**
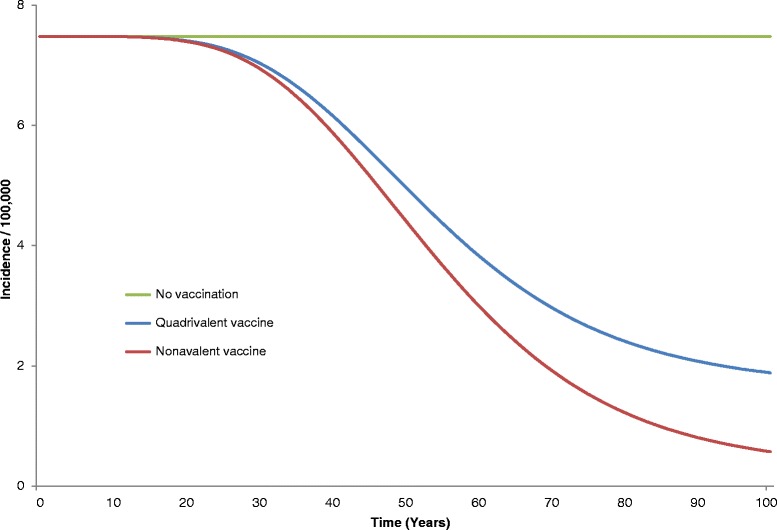
Estimated cervical cancer incidence (related to types 16, 18, 6, 11, 31, 33, 45, 52, and 58) over 100 years

The public health impact of universal vaccination on cervical cancer incidence is shown in Fig. 2. At 100 years, the nonavalent vaccine is estimated to reduce cervical cancer incidence by 92 %, corresponding to an additional 17 % decrease compared to the strategy of vaccination by Gardasil®.

The 92 % decrease in cervical cancer incidence corresponds to 13,603 cervical cancers and 5937 deaths averted compared to no vaccination over 100 years. The incremental benefit of nonavalent vaccine over the quadrivalent one is estimated to 2544 cervical cancer cases and 1124 cervical deaths over 100 years.

**Fig. 3:**
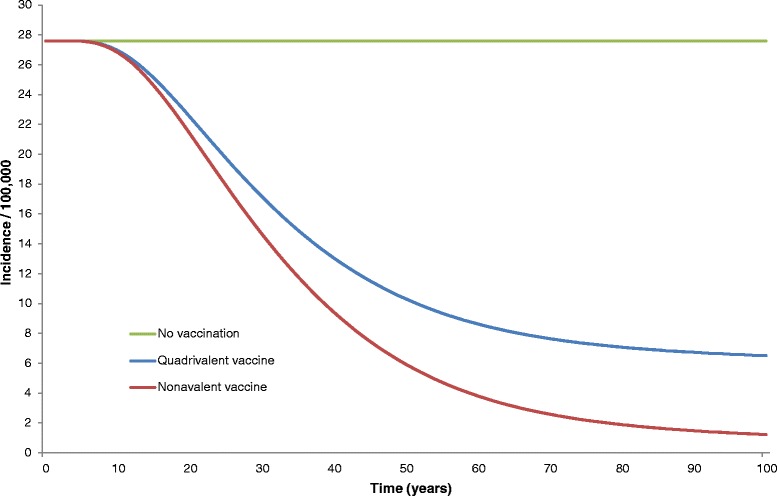
Estimated CIN2/3 incidences (related to types 16, 18, 6, 11, 31, 33, 45, 52, and 58) over 100 years

Incidences of precancerous lesions of the cervix (Cervical Intraepithelial Neoplasia 2/3) are estimated to be reduced by 96 % with the nonavalent vaccine, corresponding to an additional 20 % decrease compared to the strategy of vaccination by the quadrivalent vaccine. The nonavalent vaccine could avert an additional 14,893 cervical precancerous lesions over 100 years, compared to the quadrivalent, over 100 years (Fig. 3).

Nonavalent vaccination has an earlier effect on precancerous cervical lesion incidence reduction than for cancers. Indeed, an important burden reduction of precancerous lesion incidences is noticeable after 20 years. The incidence reductions by disease types are summarized in Table 6.

**Table 6:** Incidence reduction with quadrivalent and nonavalent vaccination at steady-state

	4-valent vaccination	9-valent vaccination
Female		
Cervical incidence	−75 %	−92 %
Cervical death	−74 %	−91 %
Genital warts	−85 %	−85 %
CIN 1	−74 %	−96 %
CIN 2+	−76 %	−96 %
Vaginal incidence	−91 %	−91 %
Vulvar incidence	−92 %	−92 %
Anal incidence	−80 %	−83 %
Anal death	−78 %	−81 %
H & N incidence	−81 %	−81 %
Male		
Anal incidence	−76 %	−78 %
Anal death	−74 %	−76 %
Genital warts	−79 %	−79 %
Penile incidence	−54 %	−55 %
H & N incidence	−77 %	−77 %

### Cost-effectiveness

**Fig. 4:**
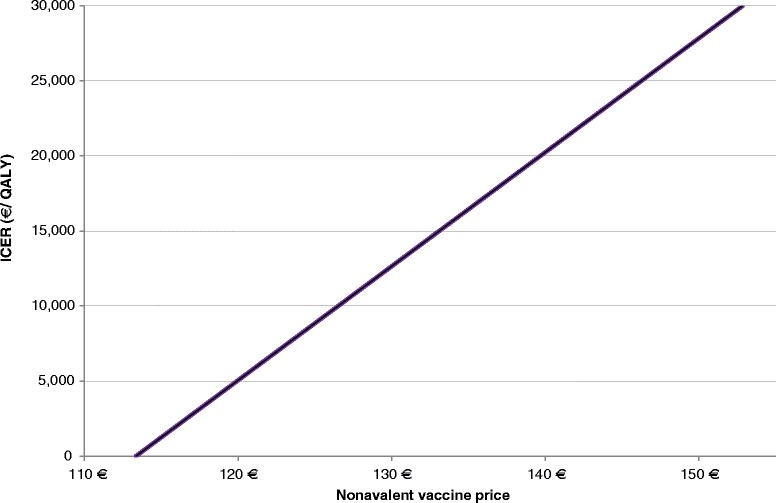
Price sensitivity

Vaccinating one cohort of Austrian girls and boys aged 9 with the nonavalent vaccine, was cost-saving at a vaccine price up to 113 € (+3€ vs the quadrivalent vaccine) and still cost-effective up to a price of 153 € (+43 €), assuming a cost-effectiveness threshold of 30,000 €/QALY gained, compared to the quadrivalent vaccine at a price of 110 € (Fig. 4). To note, should the quadrivalent vaccine price change, the vaccine price increments allowing the nonavalent vaccine to be cost-effective (+43 €) or cost-saving (+3 €) would remain unchanged.

### Sensitivity analyses

**Fig. 5:**
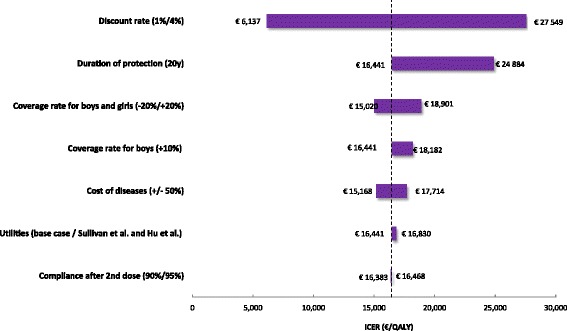
Tornado diagram of the univariate sensitivity analysis

One-way sensitivity analyses were performed to test for uncertainty are presented in Fig. 5. The base case ICER of 16,441 €/QALY gained was produced at an assumed nonavalent vaccine price of 135 € (based on US price). The costs and QALYs of the strategies tested in the base case are reported in the Table 7. The factors that considerably influenced the cost-effectiveness result, in addition to the vaccine price, were the discount rate and the duration of protection.

**Table 7:** Costs and QALY of the quadrivalent and nonavalent vaccination strategies

	4-valent vaccination	9-valent vaccination	Difference
Costs/person (€)^a^	288.44	297.18	8.74
QALYs/person^b^	26.81176	26.81229	0.00053
Cost/QALY (€/QALY)	–	–	16.441

^a^Costs rounded to 0.01

^b^QALY rounded to 0.00001

The nonavalent vaccine price has a significant impact on the ICER: universal vaccination with nonavalent vaccine was found to be cost-saving at a price of 113 € and cost-effective i.e., with an ICER < 30,000 €/QALY gained up to a price of 153 € compared to the quadrivalent vaccination.

Whichever the parameter that was varied, universal vaccination with the nonavalent vaccine remained below 30,000 €/QALY gained compared with universal vaccination with Gardasil®, ranging from 16,441 € to 28,065 €/QALY gained. Varying utilities [64, 65], cost of diseases and coverage have shown a moderate influence. An increased vaccination coverage in boys tended to increase the ICER.

## Discussion

The present study assessed the cost-effectiveness of nonavalent vaccination in the Austrian setting by adapting a dynamic model originally designed for the US. The adaptation was achieved through collection and selection of the most appropriate data from a number of countries to reflect the current Austrian epidemiological, medical and economical context, as closely as possible.

The analyses showed that nonavalent vaccination would have a great impact on the burden of HPV-related diseases in Austria. Assuming a vaccination coverage of 60 % and 40 % respectively in girls and boys aged 9, the nonavalent vaccination would reduce cervical cancer incidence related to the 9 HPV types by 92 %, CIN 2+ by 96 %, genital warts by 85 % for girls and 79 % for boys, and anal cancer incidence by 83 % and 78 % for girls and boys over 100 years, respectively compared to a no vaccination strategy. Overall universal vaccination with nonavalent vaccine would avert 23,652 cancer cases and 8399 cancer deaths, over 100 years compared to no vaccination. Compared to Gardasil®, the analyses showed nonavalent vaccine has a substantial impact on cervical cancer and precancerous lesions incidences with an incremental decrease of 18 % and 22 %, respectively, corresponding to 2544 cervical cancers and 23,174 precancerous lesions cases averted over 100 years. The benefits on anal cancer appeared to be less pronounced. This is easily explained since only 2.4 % of anal cancers are attributed to HPV types 31,33,45,52,58 whereas types 16 and 18 already included in Gardasil® are responsible for 76,3 % of the anal cancers. The current analysis demonstrates that even if the coverage rates are relatively low, especially for boys, nonavalent vaccination program has an important impact on public health. Interestingly, concrete benefits of the nonavalent vaccination come early thanks to the strong effect on genital warts and precancerous lesions. Indeed, the model estimates that as early as 20 years after the beginning of the vaccination program, incidence of genital warts and CIN2+ in females is decreased respectively by 58 % and 23 %, which is also significant from an economic perspective as at year 20, the costs averted for genital warts and CIN2+ represent 22 M€ and 77 % of the total costs averted at this time. Moreover, these estimates are likely to be very conservative, since literature reported real world-impact of the quadrivalent vaccination comes even faster with a dramatic decline in genital warts incidence observed in the first 2 years following the vaccination program implementation [66, 67] that led to a nearly disappearance of genital warts in the targeted cohorts as soon as 4 years after the commencement of the vaccination program in Australia [68]. In the same country, a decline in CIN2+ by 54 % as soon as 7 years after the implementation of the vaccination program was also reported [69].

The present analysis showed that universal vaccination with 9-valent vaccine is cost-saving up to a price of 113 € and cost effective up to a price of 153 €, in comparison to universal vaccination with quadrivalent vaccine at a price of 110 €, assuming cost-effectiveness threshold of 30,000 €/QALY gained. With a theoretical nonavalent vaccine price assumed of 135 €, vaccinating girls and boys aged 9 would be very cost-effective, compared to the current strategy, with an ICER of 16,441 €/QALY gained.

Univariate sensitivity analyses were conducted to assess uncertainty related to discount rate, vaccine’s duration of protection, vaccination coverage rates, disease costs, and QALYs. Discount rates and duration of protection were the most influential factors. However, whichever the parameter that was varied, universal vaccination of one cohort of girls and boys aged 9 remained very cost-effective compared with the vaccination strategy with the quadrivalent vaccine. These economic results can hardly be put in perspective since it is the first economic evaluation of the nonavalent HPV vaccine in Austria. However, compared to the existing economic evaluation conducted in North America, our results seems more conservative since results of the three US models concluded that - assuming comparable vaccination coverage rates than in the present study (62 % and 38 % coverage by 17 years of age) - universal nonavalent vaccination was cost-saving compared to quadrivalent universal vaccination in the US considering an incremental price per dose of 10 % (+13 US$) [23].

The current analysis has several limitations that must be presented. First, the model involved numerous parameters and not all the needed parameters could be found from Austria-specific studies. However, non-Austrian specific values have been validated by experts. Second, the cervical intraepithelial neoplasia incidences are substantially under-estimated by the model. Indeed, while the incidence of CIN2+ related to the serotypes 16,18,31,33,45,52,58 was estimated to be 114 / 100,000, the model estimates 27.62 / 100,000 corresponding to a 5-fold underestimation. Furthermore, the additional benefits of nonavalent vaccination on CIN may also be underestimated. In the study from Hartwig et al. [4], results showed that the HPV6/11/16/18 are responsible for about 45 % of CIN2+, whereas HPV6/11/16/18/31/33/45/52/58 targeted by the nonavalent vaccine account for 82 % of CIN2+ meaning the nonavalent HPV infections were responsible for 1.8 times more CIN2+ compared the quadrivalent HPV whereas the results obtained from the calibrated model indicated that nonavalent HPV accounts for 1.24 times more for CIN2+ than quadrivalent HPV, minimizing greatly the benefit of the nonavalent vaccine on CIN compared to the quadrivalent. Overall, although nonavalent vaccination was estimated to bring important benefits on CINs, the latter are dramatically underestimated by our model. The inability to calibrate appropriately on the CIN is explained by the natural history parameters of the model that do not allow us to have a good fit on both cervical and CIN incidence. The choice was made to privilege the cervical cancer calibration since it is the most impactful parameter. This underestimation constitutes a limit of the model that must be fixed in the future. Nevertheless, the benefits of nonavalent vaccination being underestimated, our analyses remain conservative. Likewise, vaginal and vulvar incidences are under-estimated. These model limitations should be corrected in the future but led to increased –and consequently conservative - ICER estimate for the nonavalent vaccination strategy. Third, the model assumes disease attribution for the HPV types 31,33,45,52,58 only for cervical and anal diseases. Benefit of the nonavalent vaccine on vulvar and vaginal cancers are consequently underestimated in our analyses. However, the vulvar and vaginal cancer incidence being far lower than cervical cancer incidence, this limitation should marginally impact the analysis results. Fourth, the economic benefits of HPV vaccination are underestimated since the indirect effects of cancer (loss of income for patients due to disruption in professional life, indirect costs for childcare or caregiver costs, increase of private insurance cost…) are not considered in this analysis. Fifth, the model focused on heterosexual transmission of HPV and did not incorporate transmission between men who have sex with men (MSM), or between homosexuals and heterosexuals. Last, the model does not consider the population movement (immigration and emigration).

It must be emphasized that by expanding the spectrum of prevention to 80–90 % of HPV-related cancers and other diseases, the nonavalent vaccine - should the coverage rates be high enough – contribute to reframe the cervical cancer prevention model shifting towards a more comprehensive HPV prevention model. Indeed, as nonavalent vaccination will further lower the prevalence rates of cervical lesions, the performance (positive predictive value, PPV) of Pap cytology test will decrease. Pap cytology test performance falls dramatically at lesion rates <20 %. With further reduction in lesions prevalence consequent to HPV vaccination (<5 %), the clinical utility of the PPV becomes substantially affected with the implication being that the vast majority of cases identified on screening will result in unnecessary clinical management and follow-up and attendant higher costs [70, 71]. This paradigm shift is already observed in the Netherlands where the HPV DNA test should be implemented as first line screen for cervical cancer prevention in 2016. Furthermore, it has to be highlighted the societal impact of nonavalent vaccination goes far beyond the epidemiological outcomes reported above. Indeed, the epidemiological change induced by the nonavalent vaccine translates notably in less emotional suffering linked to screening outcomes (fear about the future and potential fertility concerns, possible lifelong disability post-surgery, etc.…) and it preserves fertility by avoiding potential adverse pregnancy outcomes post cervical therapy (pre-term birth increased by 2 to 3 after conisation) [72]. These effects on patient’s lives are not taken into account in the evaluation but are far to be negligible [73].

## Conclusion

The results of the present evaluation show that vaccinating 60 % of girls and 40 % of boys aged 9 in Austria with a nonavalent vaccine will substantially reduce the incidence of cervical cancer, CIN and anal cancer by 20 %, 17 % and 3 % respectively compared to the existing strategy. The vaccination strategies performed with the nonavalent vaccine in the current study were all found to be cost-effective compared to the current quadrivalent vaccination strategy by considering a cost-effectiveness threshold of 30,000€/QALY gained.

The nonavalent vaccine has been considered an important advance over Gardasil®, as the nine vaccine types account for about 90 % of cervical cancer. The present study demonstrate that the switch from Gardasil® to the nonavalent universal vaccination in Austria can bring substantial incremental public health benefits and would constitute a cost-effective intervention.

### Ethics approval and consent to participate

Not applicable. The study does not involve human subjects.

### Consent for publication

Not applicable. The article does not include any material relating to individual participant.

### Availability of data and materials

All data supporting our findings will be shared upon request.

## Abbreviations

CEA: cost-effectiveness analysis
CHMP: Committee for Medicinal Products for Human Use
CIN: cervical intraepithelial neoplasia
CIS: carcinoma in situ
HPV: Human Papillomavirus
ICER: incremental cost-effectiveness ratio
MSM: men who have sex with men
NATSAL: National Survey of Sexual Attitudes and Lifestyles
Pap: papanicolaou smear
QALY: quality-adjusted life year
QoL: quality of life
RRP: recurrent respiratory papillomatosis
VaIN: vaginal intraepithelial neoplasia
VIN: vulvar intraepithelial neoplasia

## Footnotes

1 * Considering 1 USD = 0.906849 EUR (www.xe.com, visited the 28^th^ of July 2015)
